# The Potential of Physical Exercise to Mitigate Radiation Damage—A Systematic Review

**DOI:** 10.3389/fmed.2021.585483

**Published:** 2021-04-29

**Authors:** David S. Kim, Tobias Weber, Ulrich Straube, Christine E. Hellweg, Mona Nasser, David A. Green, Anna Fogtman

**Affiliations:** ^1^Space Medicine Team (HRE-OM), European Astronaut Centre, European Space Agency, Cologne, Germany; ^2^Faculty of Medicine, University of British Columbia, Vancouver, BC, Canada; ^3^KBR GmbH, Cologne, Germany; ^4^Radiation Biology Department, Institute of Aerospace Medicine, German Aerospace Centre (DLR), Cologne, Germany; ^5^Peninsula Dental School, Plymouth University, Plymouth, United Kingdom; ^6^Centre of Human & Applied Physiological Sciences (CHAPS), King's College London, London, United Kingdom

**Keywords:** ionizing radiation, deep space exploration, human spaceflight, radiation countermeasures, physical exercise, DNA damage, oxidative stress

## Abstract

There is a need to investigate new countermeasures against the detrimental effects of ionizing radiation as deep space exploration missions are on the horizon.

**Objective:** In this systematic review, the effects of physical exercise upon ionizing radiation-induced damage were evaluated.

**Methods:** Systematic searches were performed in Medline, Embase, Cochrane library, and the databases from space agencies. Of 2,798 publications that were screened, 22 studies contained relevant data that were further extracted and analyzed. Risk of bias of included studies was assessed. Due to the high level of heterogeneity, meta-analysis was not performed. Five outcome groups were assessed by calculating Hedges' *g* effect sizes and visualized using effect size plots.

**Results:** Exercise decreased radiation-induced DNA damage, oxidative stress, and inflammation, while increasing antioxidant activity. Although the results were highly heterogeneous, there was evidence for a beneficial effect of exercise in cellular, clinical, and functional outcomes.

**Conclusions:** Out of 72 outcomes, 68 showed a beneficial effect of physical training when exposed to ionizing radiation. As the first study to investigate a potential protective mechanism of physical exercise against radiation effects in a systematic review, the current findings may help inform medical capabilities of human spaceflight and may also be relevant for terrestrial clinical care such as radiation oncology.

## Introduction

Ionizing radiation (IR) has been shown to pose significant risks to living organisms by causing damage at tissue and molecular levels. In cancer therapy, IR is used to target cancerous cells, while attempting to limit damage to nearby healthy cells and reducing the incidence of secondary cancers (1). Thus, methodologies to decrease secondary damage and implementing new models of bio-protection from the harmful effects of IR could significantly improve patient outcomes. In fact, IR greater than baseline levels has been proposed to cause ~10% of all cancer incidence (2). As a result, there has been a drive to better understand how radiation affects living organisms and the mechanisms that underlie these effects in order to innovate protection or countermeasures against the negative effects of IR (3).

IR induces biological damage due to energy transfer when it interacts with tissues (4). IR can directly harm the genome by causing DNA damage via the formation of single- and double-strand breaks (SSB and DSB), replication stress (checkpoint activation), and more complex oxidative clustered DNA damage (OCDLs) (5, 6). Unless the DNA damage is repaired by standard mechanisms, the risk of cancer increases as the probability of mutations and its downstream oncogenic potential increases (7). There are also indirect effects of IR caused by free radicals and reactive oxygen species (ROS) formed via radiolysis of water molecules resulting from absorption of energy. Free radicals and ROS represent cellular oxidative stress, which can lead to DNA damage and result in deleterious genomic instability characterized by accumulation of chromosomal aberrations (8).

Radiation effects on DNA molecules are termed targeted effects, whereas those that occur in non-irradiated tissues or in non-directly hit cells are called non-targeted effects (NTEs) (9). NTEs may be more important at lower doses, in particular radiation-related inflammation (10, 11). In fact, it is probable that inflammatory mediators play a significant role in eliciting NTEs and can induce further DNA cluster formations (12). All such responses encompass the activation of several signaling pathways and potentially long-lasting changes in gene expression that may cause tissue damage or cancer (13).

IR has also been identified as the main hazard to human exploration of deep space (4), which must be considered in space mission design with respect to extremely limited medical capabilities of spaceflight and the complex nature of space radiation (14). Without the protection of the Earth's atmosphere and the Earth's electromagnetic shield, exposure to the densely ionizing Galactic Cosmic Rays (GCR) is estimated to be up to 770 times of that on Earth (Cologne, Germany) and 250 times higher than on the International Space Station (ISS) (15–17). Thus, beyond Low Earth Orbit (LEO), the risks associated with IR may even be prohibitive for deep space flight missions without sufficiently effective shielding or countermeasures (4). For both space and Earth, more research is required on the effects of IR and methodologies to protect living organisms.

Interestingly, aerobic exercise has been shown to reduce all-cause mortality via lowering the risk of cardiovascular, endocrine, musculoskeletal, immunological, and oncological disease (18). While acute aerobic exercise can induce oxidative stress in the immediate period (19), repeated longer exercise induces protective cellular adaptations (20, 21). These include mitochondrial biogenesis and increased oxidative phosphorylation capacity in addition to a range of positive downstream signaling cascade effects (22). Evidence of increased resilience to oxidative stress, DNA damage, and inflammation has been reported in response to exercise (18, 22). In addition, contracting muscles release myokines that improve tissue regeneration, repair, immunomodulation, and cell signaling (22). Recently, at a macro level, the cumulative molecular and biochemical effects of longer-term aerobic exercise training has been shown to correlate to cardiorespiratory fitness with improved survival without an upper limit of benefit in a large trial of 122,007 patients (23).

In addition to the direct and indirect effects of space radiation encountered, space-relevant radiation qualities are also very efficient in activating the pro-inflammatory transcription factor Nuclear Factor κB (NF-κB) and its target genes that encompass proinflammatory cytokines (24–26). Thus, as exercise initiates acute transient oxidative stress states that lead to protective long-term anti-inflammatory and other potential molecular radio-protective effects, these beneficial adaptive mechanisms induced by exercise could also potentially counteract the negative effects of IR exposure. Furthermore, similar benefits may have important implications for terrestrial medicine including cancer therapy and radiation protection (27, 28).

However, to our knowledge, no systematic review has evaluated the potential radio-protective effects of exercise training. Thus, the present study evaluated the evidence for, and candidate mechanisms underlying potential radio-protective effects of exercise. Therefore, clinical, functional, DNA damage/oxidative stress/inflammation, neurogenesis, and cellular/tissue function outcomes were investigated in the present study. The current findings may be of relevance not only in space travel but also for terrestrial applications in the context of radiation oncology, occupational radiation exposure, accidental radiation exposure, and public health.

## Methods

### Search Strategy

A systematic search strategy was designed and performed in electronic databases on September 2019 (29). Databases used in the search were Medline, Embase (OVID), CENTRAL (Cochrane library), and the databases from space agencies: European Space Agency (ESA), National Aeronautics and Space Administration (NASA), and the German Aerospace Center (DLR - Deutsches Zentrum für Luft- und Raumfahrt). The search strategy is composed of terms for radiation, combined with those for exercise using Boolean operators, Medical Subject Headings **(MeSH)** Terms, and a combination of search terms and subject headings, where available. Details of the search strategy are given in Supplementary Table 1. The strategy for searching the space agencies' databases was simplified by using main keywords from each of the radiation and exercise concepts.

### Criteria for Selection

The protocol of the review is available on request from the authors. The PICOS (Population, Intervention, Control, Outcomes, and Study design) question framework was used to inform the selection criteria reproduced below:

P—Humans, animals, and cell lines (terrestrial and/or space)
I—Exercise (aerobic, resistive, other) prior to, during, or after partial or whole-body exposure to ionizing radiation
C—No intervention
O—Clinical or molecular/biochemical/cellular responses to radiation exposure
S—Experimental studies.

### Study Selection and Extraction of Data

**Figure 1:**
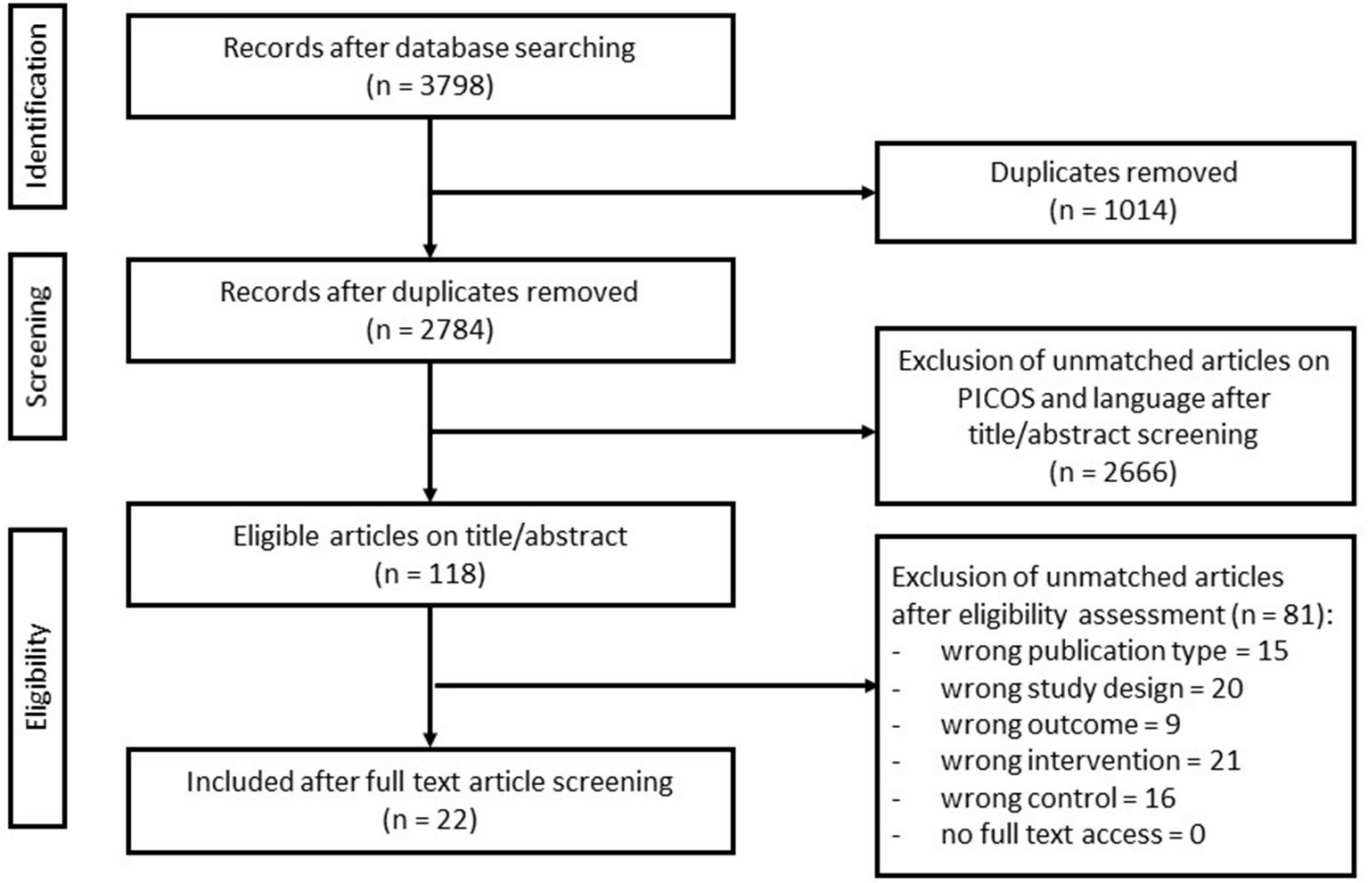
Flow diagram of search and screening methodology. The flow of search results, numbers, and author's eligibility screening assessment is represented here.

All studies were imported into the Rayyan Software (Web Rayyan QCRI) (30) and screened by at least two qualified reviewers using the described inclusion criteria. The initial screen excluded studies based on assessment of title and abstract. Full texts of the remaining articles were obtained and assessed against the inclusion criteria. Any discrepancies of opinion were discussed with a third expert reviewer. Resolution of discrepancies between the three reviewers was achieved through consensus. Exclusion criteria were other adjunct interventions (such as chemotherapy, hormone therapy, and biological therapy), lack of control group, and studies not matching the PICOS. The process of inclusion and flow diagram of studies are listed in the **Results** section and in Figure 1.

### Data Extraction and Reporting

From all the final included studies, data according to the outcomes were extracted using a modified version of the Cochrane's EPOC Good Practice Data Extraction Form (31). Data extracted include study design, type, characteristics, population, exercise (intensity, duration, type, and chronology), radiation (dose, duration, type, target, and chronology), statistical methods employed, and results. Outcomes were categorized into one of the five groups: DNA effects (DNA damage/oxidative stress/inflammation), neurogenesis, functional, cellular function, and clinical. The Preferred Reporting Items for Systematic Reviews and Meta-Analyses (PRISMA) checklist was used to aid in the reporting process (32).

### Quality of Included Studies

All studies were controlled prospective studies in animals and humans except for one that was a cohort study (21), which was analyzed separately. All controlled studies were appraised in accordance with Cochrane's risk of bias methodology (29). Studies were scored with “?” representing unclear risk, “+” representing low risk, and “-” representing high risk of bias. Any uncertainties were discussed with two reviewers at minimum and assessed to find a consensus.

### Data Analysis and Statistics

The studies and their outcomes were very heterogeneous; therefore a standard meta-analysis was not possible. Thus, for all outcomes, effect sizes were calculated using the Hedges' ***g***value corrected and plotted on an effect size plot for each grouping of outcomes using the mean and standard deviation value (33). For studies that reported standard error (SE), it was converted to standard deviation (SD) using the formula: SD = SE × √N. For studies that reported results only in chart or figure form, a plot reader (WebPlotDigitizer) was utilized to extract the raw data (34). A 95% confidence interval was set when deriving the effect sizes for comparison of all outcomes. Due to lack of studies on minimal important differences for the outcomes in this context, the following guidance to categorize the magnitude of effect sizes was used to inference its probability of having a true effect, where 0.1 was small, 0.3 was moderate, 0.5 was large, 0.7 was very large, and 0.9 was extremely large in its comparison between intervention and control groups (33).

Some outcome values increase or decrease in its effect in response to the intervention but this may not necessarily correlate to a beneficial or no beneficial effect (for example, exercise training may decrease resting heart rate, which is a beneficial effect but gives a negative effect size). Thus, for those studies, the mean values of the outcomes were multiplied by −1 ensuring all scales in the effect size plots follow the logic that a positive rightward direction indicates a beneficial effect in favor of the exercise intervention group. Conversely, the plots that are in the negative leftward direction indicate a beneficial effect in favor of the control groups that did not exercise. Collected data and associated metadata were assessed to analyze relationships between experimental conditions and outcomes using SankeyMATIC tool (35) and “Charticulator” (36). The low number of studies with similar designs were not sufficient to conduct a funnel plot to assess publication bias.

## Results

### Search Results and Study Characteristics

The search query yielded 3,798 studies, of which 1,014 were identified as duplicates. The remaining 2,784 studies were screened using its title and abstract, resulting in the exclusion of 2,666 studies as they did not meet the inclusion criteria. The full text was retrieved of the remaining 118 studies and screened, yielding 22 final studies. Reasons for exclusion included inappropriate publication type, study design, outcome, intervention, or controls (Figure 1).

Of the final 22 included articles, nine reported data from human subjects, and the remaining 13 were experimental animal studies. The included studies were published between 2002 and 2019. In addition, the reference lists of all 22 included studies were obtained and screened in an attempt to identify any studies that may not have been included in the original search, but none were deemed eligible.

The radiation dosage and delivery modality in the included studies were diverse with a combination of photons (γ-rays and X-rays), electrons, protons, or high-atomic-number (Z) and high-energy charged (HZE) particles such as carbon ions with varying degrees of exposure (whole body and partial, including brain or tumor directed) with doses ranging from 1 to 20 Gy and 50 to 76 Gy depending on the study type and studied genus (Table 1). In addition, the time at which the exercise intervention was performed with respect to irradiation varied with five studies before (Pre), six during (During), and 11 after exposure to IR (Post). Exercise interventions were mostly aerobic, with four studies being a mix of aerobic and resistance, and one study employing only resistance training. The duration, intensity, and progression of exercise training interventions were heterogeneous. In total, 74 different outcomes (72 with calculated effect sizes and two without) were reported across the 22 studies, including 15 human and 59 rodent outcomes (Supplementary Table 2).

**Table 1:** Irradiation conditions and outcome types in studied articles.

**Irradiation area**	**Genus**	**Radiation quality**	**Radiation dose**	**No. of publications**	**Outcome type**
Whole Body	Animals	Photons (γ-rays, 0.6617–1.25 MeV)	1.116–3.5 Gy	4	Cellular, DNA Effects, Neurogenesis
		Photons (X-rays, 6 MeV/n)	10 Gy	1	Neurogenesis
		HZE (Carbon ions, 290 MeV, 42.35 keV/μm)	1–5 Gy	1	Cellular
Brain	Animals	Electrons (4 MeV)	20 Gy	1	Functional, Neurogenesis
		Photons (γ-rays, 1.25 MeV)	3.5 Gy	1	Neurogenesis
		Photons (X-rays, 0.35–6 MeV)	5–10 Gy	4	DNA Effects, Functional, Neurogenesis
		Photons^*^ (X-rays)	20 Gy	1	Functional, Neurogenesis
Tumor	Humans	Photons (X-rays, 6–18 MeV)	50–70 Gy	2	Functional
		Photons^*^ (X-rays)	n/a	1	Functional, DNA Effects
		Photons^*^	52 Gy	1	Clinical
		n/a	50–76 Gy	3	Functional, DNA Effects
		n/a	n/a	2	Functional, Clinical

* *No information provided on radiation quality and/or dose*.

**Figure 2:**
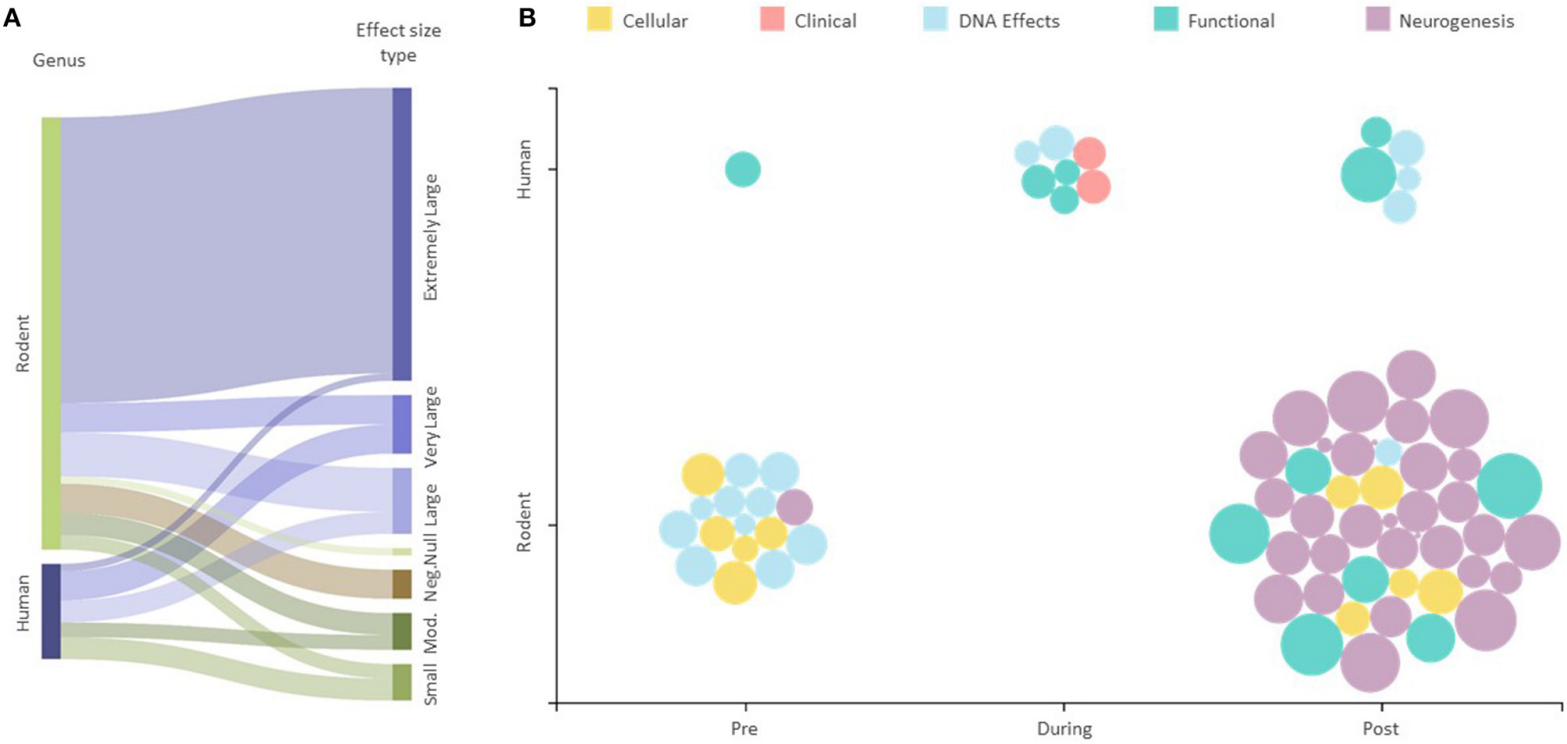
Effect of exercise intervention explained by experimental conditions. **(A)** Sankey diagram showing the effect sizes for all human and animal studies. The string thicknesses refer to the total number of outcomes for each category. “Mod.” —Moderate, “Neg.”—Negative, “Null “—No effect. **(B)** Effect size scatter plot showing all human and animal outcomes. Pre, During, and Post refer to the time at which the exercise intervention was applied with respect to the radiation exposure. The size of bubbles represents the effect sizes for each outcome represented.

The majority (40 or 55.6%) of the assessed 72 outcomes had extremely large effect sizes that were in favor of the intervention groups (Supplementary Table 2). There were eight (11.1%) outcomes with very large effect sizes, nine (12.5%) with large effect sizes, five (6.9%) outcomes with a moderate effect size, and five with small effect sizes, all in favor of the intervention groups. Four of the 72 (5.5%) outcomes were reported as negative, and one showed no effect (vs. control) (Figure 2A). The majority (59 out of 72) of the reported outcomes were from rodents that exercised before and after exposure to IR, with 31 of these being neurological, and 13 of 72 outcomes were from human studies (Figure 2B).

### Evidence of Risk of Bias

Studies were scored based on their quality and bias (Supplementary Table 3). Most of the included studies had insufficient data and/or failed to report elements to allow a full assessment of bias. For example, many articles did not report a specific randomization method even though it was stated that participants were randomized. Four studies were assessed as possessing a high risk of bias, and the rest were classified as having an unclear risk of bias.

### Effect of Exercise Training on DNA Effects of IR

**Figure 3:**
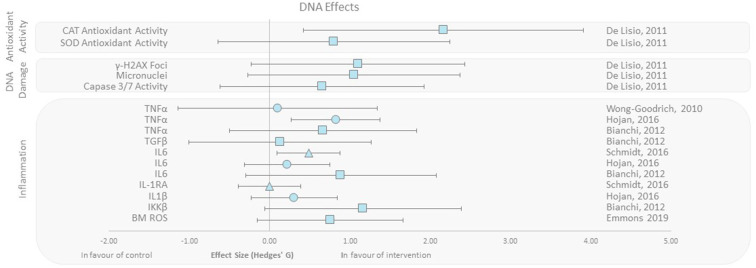
Effect size plot of antioxidant activity, DNA damage, and inflammation. Outcomes are plotted with the Hedges' *g* calculated for each outcome extracted and bias corrected for sample size. Confidence intervals of 95% are represented by the error bars. Effect size values that are in the positive rightward direction indicate a beneficial effect in favor of the exercise intervention group compared to the non-exercise control group. Shapes refer to the time at which the exercise intervention was applied with respect to the radiation exposure (Post—Circle, Pre—Square, During—Triangle).

DNA damage, oxidative stress, inflammation, and antioxidant activity outcomes were grouped as DNA effects. De Lisio et al. (37) found that superoxide dismutase (SOD) and catalase (CAT) antioxidant activity increased with effect sizes of 0.8 and 2.15, respectively, in a (pre-γ-ray exposure) exercised group of mice vs. no exercise control. Additionally, studies presenting inflammatory markers when exposed to radiation found that IL-1, IL-6, TNF-α, and TGF-β levels decreased in the intervention group that exercised. Emmons et al. (27) also reported that progressive treadmill exercise in mice before exposure to γ-rays decreased bone marrow ROS levels (effect size 0.75). Studies reporting DNA damage with respect to micro-nucleation, Caspase 3/7 activity, and γH2AX foci indicating DNA DSBs all favored the intervention group with effect sizes ranging from 0.65 to 1.10 (Figure 3) (27, 37–42).

### Effect of Exercise Training on Radiation-Induced Neurogenesis Changes

**Figure 4:**
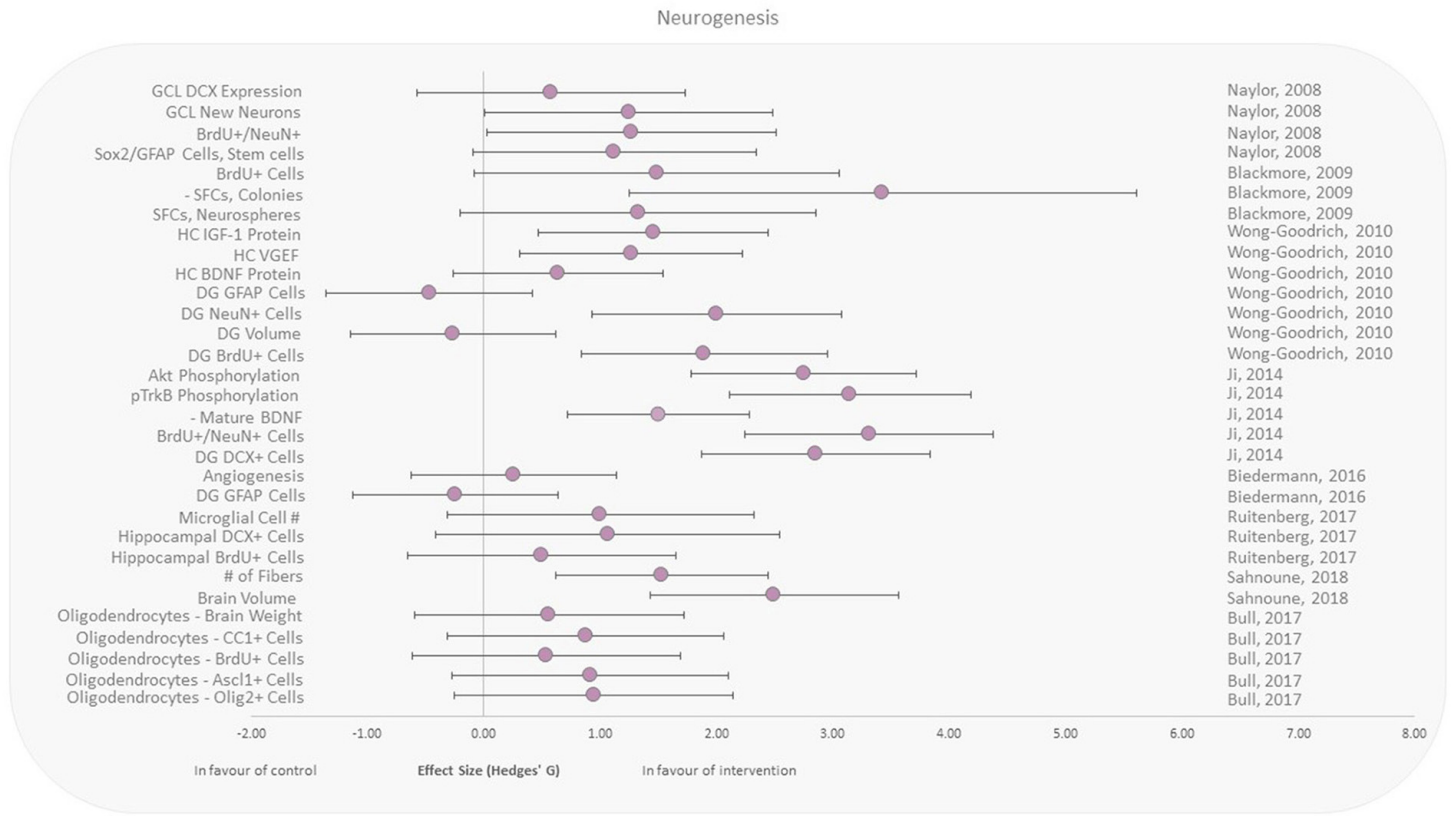
Effect size plot of neurogenesis in animal studies. Outcomes are plotted with the Hedges' *g* calculated for each outcome extracted and bias corrected for sample size. Confidence intervals of 95% are represented by the error bars. Effect size values that are in the positive rightward direction indicate a beneficial effect in favor of the exercise intervention group compared to the non-exercise control group. Shapes refer to the time at which the exercise intervention was applied with respect to the radiation exposure (Post—Circle).

Neurogenesis was assessed following both brain and whole-body irradiation. All included studies here reported responses to either X-rays, γ-rays, or electrons in animals that performed aerobic exercise after irradiation (Figure 4) (39, 43–49). Included studies reported beneficial effects of running upon cell proliferation and angiogenesis in the hippocampal region (vs. control), independent of the irradiation area. Expression of the astrocyte intermediate filament glial fibrillary acidic protein (GFAP) in the dentate gyrus (DG) was reduced in two murine studies, with moderate effect sizes of −0.24 and −0.46 (39, 46). Wong-Goodrich et al. (39) also reported reduced (effect size −0.26) dentate gyrus volume with voluntary wheel running post-X-ray exposure compared to control. Two other recent studies reported brain volume and weight increases (48, 49).

### Effect of Exercise Training on Radiation-Induced Changes in Functional Outcomes

**Figure 5:**
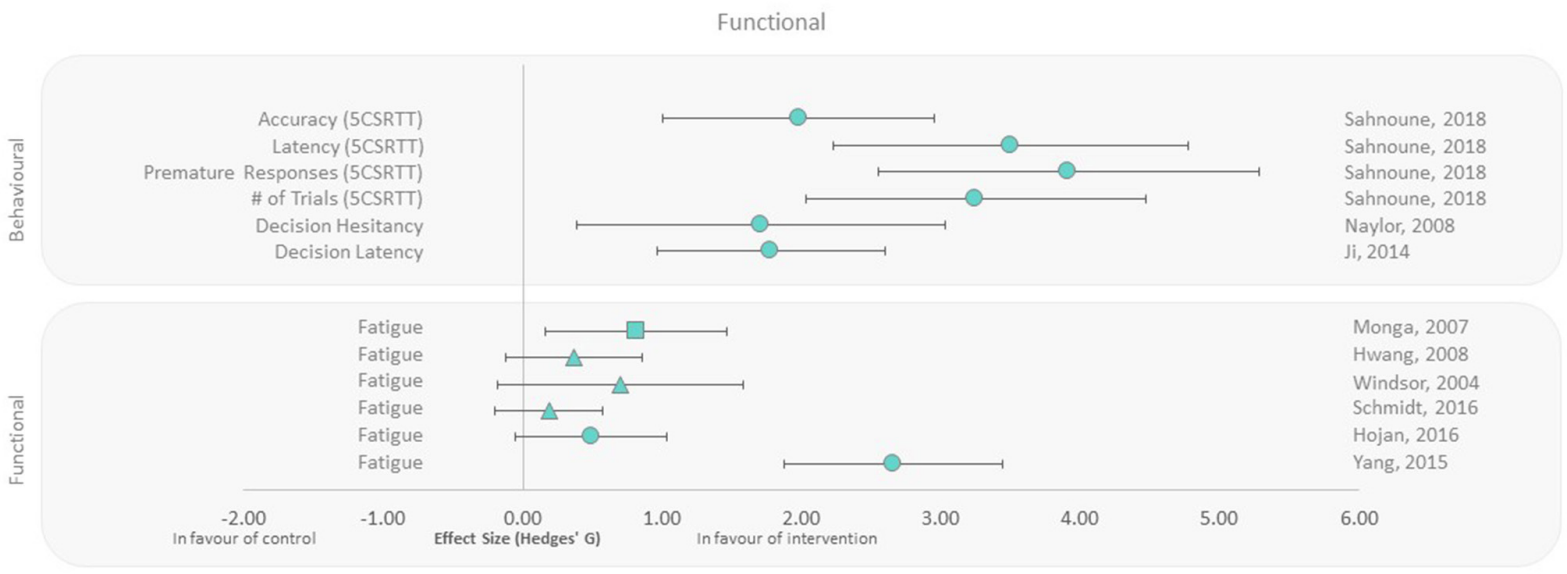
Effect size plot of behavior in animals and fatigue in human trials. Outcomes are plotted with the Hedges' *g* calculated for each outcome extracted and bias corrected for sample size. Confidence intervals of 95% are represented by the error bars. Effect size values that are in the positive rightward direction indicate a beneficial effect in favor of the exercise intervention group compared to the non-exercise control group. Shapes refer to the time at which the exercise intervention was applied with respect to the radiation exposure (Post—Circle, Pre—Square, During—Triangle).

Functional and behavioral outcomes were reported in human and animal studies, respectively (40, 42, 43, 45, 48, 50–53). All rodent behavioral outcomes (decision making, latency, accuracy, and hesitancy) were beneficial in the exercise groups compared to controls, with extremely large effect sizes ranging from 1.71 to 3.92 (Figure 5). Fatigue as a functional outcome was assessed in female and male patients undergoing radiotherapy. All studies reported lower fatigue irrespective of when the exercise intervention was applied (Pre, During, and Post), compared to the control groups, with effect sizes ranging from 0.19 to 2.66. The largest effect size was observed with 7-week aerobic exercise training performed after irradiation (Figure 5). Conversely, the smallest effect size was observed in the only study that applied resistance training, despite it representing the longest exercise duration among all human studies within this outcome group (12 weeks of training during radiotherapy).

### Effect of Exercise Training on Radiation-Induced Changes in Cellular Processes

**Figure 6:**
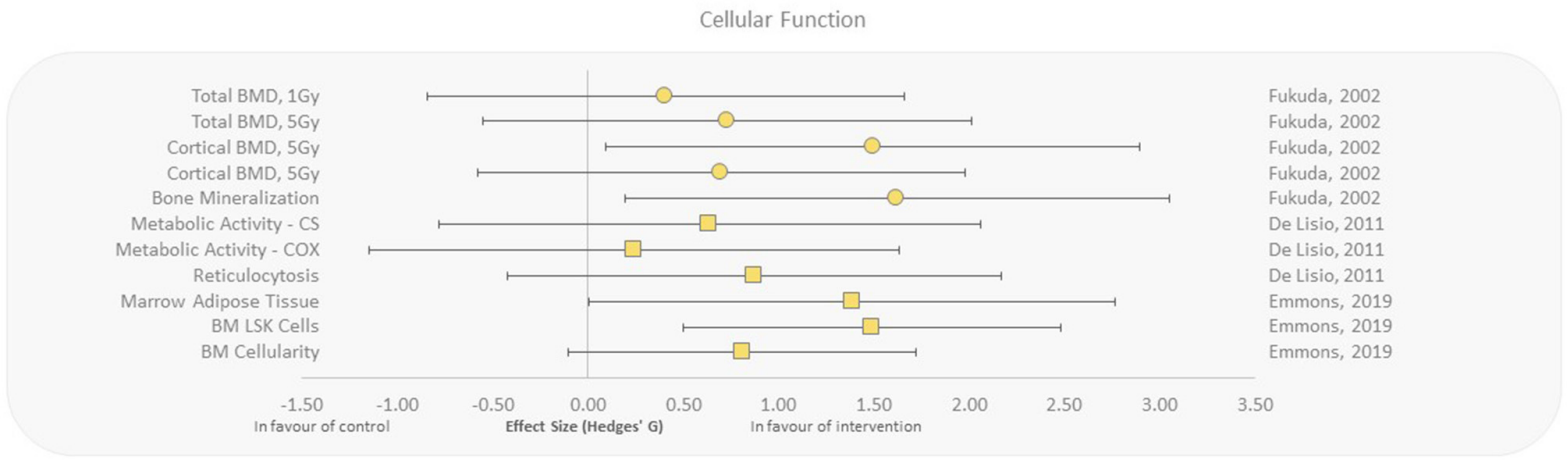
Effect size plot of cellular and tissue function in animal studies. Outcomes are plotted with the Hedges' *g* calculated for each outcome extracted and bias corrected for sample size. Confidence intervals of 95% are represented by the error bars. Effect size values that are in the positive rightward direction indicate a beneficial effect in favor of the exercise intervention group compared to the non-exercise control group. Shapes refer to the time at which the exercise intervention was applied with respect to the radiation exposure (Post—Circle, Pre—Square).

Cellular and tissue function outcomes were assessed with whole-body irradiations only, but showed effect sizes in favor of aerobic exercise with regard to bone marrow cellularity, reticulocytosis, metabolic enzyme activity, and bone mineralization/density (27, 37, 38, 54). Emmons et al. (27) reported that exercised mice had increased bone marrow cellularity, decreased marrow adipose tissue, and increased Lin^−^Sca-1^+^c-kit^+^ (LSK) cell populations as a response to exercise suggesting rescue of hematopoietic stem cells that were destructed by γ-irradiation, with effect sizes ranging from 0.81 to 1.49. Increased reticulocytosis and metabolic enzyme activity was observed when mice progressively exercised for 10 weeks after exposure to γ-rays compared to control (37, 38). Fukuda et al. studied the effect of aerobic exercise on cellular responses to HZE particles (high charge, high energy) in irradiated rats (54). They reported moderate to extremely large effect sizes for bone mineralization and density, in exposed animals that underwent 5 weeks of running after radiation exposure (Figure 6).

### Effect of Exercise on Radiation-Induced Clinical Changes

The clinical outcomes as measured by bladder and rectal toxicity were studied in male patients undergoing radiotherapy for prostate cancer. A static aerobic exercise training during prostate cancer radiotherapy resulted in lower rectal (0.71) and bladder (0.60) toxicity showing favorable outcomes for the intervention group (55).

### Missing Data

Some data that were extracted was unable to be represented on an effect size plot due to an inability to calculate a Hedges' *g* value from hazard ratios and Mann–Whitney *U* scores, respectively (Supplementary Table 4). However, both studies reported statistically significant outcomes in favor of the exercise group. Chang et al. (21) reported in a subgroup of a large breast cancer radiotherapy cohort that physical activity was linked to a reduction in acute coronary events with a hazard ratio of 0.27 (CI = 0.13–0.57; *p* < 0.001). Aghili et al. (56) performed a prospective, non-randomized parallel group trial of exercise vs. control patients undergoing radiotherapy, reporting a significant reduction in fatigue in the exercise group (Mann–Whitney *U* score of 28.5; *p* < 0.001).

## Discussion

This systematic review found highly heterogeneous studies, in the populations studied, outcomes measured, and experimental design. No included study evaluated the effect of exercise training as a countermeasure to the whole biological system. However, specific cellular, organs', or tissues' functions were reported. In both animals and humans, reported outcomes were in favor of exercise training as an intervention. Of the 72 outcomes that were extracted for this review, only three outcomes had an effect in favor of the control group, one outcome did not show a difference in the groups, and the rest of the 68 outcomes showed a beneficial effect of exercise when exposed to IR. It seems also evident from the large effect sizes across all outcome parameters that exercise may play a positive role in all biological domains with regard to improving molecular, genomic, cellular, functional, and clinical outcomes.

It was not possible to assess publication bias as the number of studies measuring the same outcomes and performing similar comparisons was insufficient. It was also not possible to assess reporting bias as the study protocol of most studies were not available. However, publication bias can be expected to a certain degree as not all internal studies of all agencies have been published in peer-reviewed journals. Although some space agencies provide publicly available databases like NASA and ESA, this is not the case for others like the Russian space agency.

### Outcome Groups

One potential explanation for the apparent radio-protective nature of exercise training could be downstream effects that increase DNA repair mechanisms and antioxidant activity and decrease inflammatory stress. Each of these mechanisms would create an environment of resistance to DNA damage and improved repair capacity, which would be highly advantageous when exposed to IR. This was in fact observed (Figure 3) with clear beneficial effects on DNA damage and response pathways including increased antioxidant activity, decreased inflammatory markers, and improved DNA repair in response to exercise when exposed to IR. We also showed that exercise causes a reduction in pro-oncogenic environments (modification of TNF, IL, micronuclei, and caspase activity) that could potentially lead to a reduction in radiation-induced cancer incidence (57). Studies evaluating biomarkers of oncogenesis, cancer incidence, and survival are warranted to define whether this is the case in humans.

Exercise has been shown in various studies to improve neurogenesis, learning, memory, and hippocampal plasticity, and to reduce natural age-related cognitive deficits (58–60). Detrimental effects of IR on brain function, neurogenesis, and cognitive function are also well known (61, 62). However, studies investigating the effect of exercise on irradiation-induced changes in the human brain are few. The selected studies in this review are all from animal models assessing the negative effects of radiation exposure of the brain (Figure 4). All the studies involved irradiation before the exercise intervention. Although the radiation doses delivered to the animals varied between 3.5 and 20 Gy, the exercise intervention was homogeneous across these studies (aerobic running wheel performed after the irradiation). These results seem to point to the beneficial effect of exercise in rescuing neurogenesis with varying levels of positive effect sizes except for three outcomes.

As reported by Wong-Goodrich et al. and Biedermann et al., the outcome in the intervention group with regard to GFAP cells in the dentate gyrus (DG) with a decrease in GFAP expression was unfavorable (39, 46). While GFAP expression in the DG decreased in these studies, GFAP expression may not be strictly correlated to neurogenesis in a monocausal relationship (63). Biedermann et al. shows that irradiation alone actually increased GFAP^+^ cells in the hippocampus rather than an expected decrease (46). Additionally, other studies have shown that GFAP expression is also a marker of gliogenesis, which suggest that the reason for GFAP increase in irradiated mice and decrease in exercise groups may be due to different etiologies (63). Decrease of GFAP expression may also be in favor of the exercise group, due to it being also a marker of inflammatory processes, glial scars, neurodegenerative diseases, and aging (64).

It should also be noted that Wong-Goodrich et al. found a decrease in the DG volume in the exercise group (39). However, studies show that DG volume or size is not directly correlated to neurogenesis and that it may actually decrease in response to exercise (65, 66). This may be explained as inflammation has been shown to increase brain volume in some regions and chronic expression of inflammatory markers such as TGFβ1 is also associated with increased volumes (66). Thus, inflammation may have been attenuated in exercise, which resulted in an overall reduction in DG volume. However, it is also possible that regional brain volume changes may fluctuate depending on the time point at which assessment is performed with relation to radiation effects/exercise performance (47).

Behavioral outcomes in mice from three studies all demonstrated a positive impact of exercise on decision-making accuracy, speed, and hesitancy (Figure 5). This is consistent with cognitive function improvements associated with neurogenesis and hypothalamic biofunction, induced by exercise (39, 49). Although the animal cognitive function methodologies varied between studies, they all suggest improved cognitive ability with exercise, even if performed following irradiation. Further correlates are required to make similar conclusions in humans in the context of cranial radiotherapy and cognitive function. The functional outcome reported in radiotherapy patients (Figure 5) was fatigue; however, there was heterogeneity in study design including differing types of exercise interventions. Neither the dose nor the timing of radiotherapy appeared to influence reductions in fatigue observed in the exercise intervention group.

Only a single selected study reported clinical outcomes specific to humans, where bladder and rectal toxicity was lower in patients undergoing radiotherapy for prostate cancer with aerobic exercise (55). A number of articles reporting human trials of patients undergoing radiotherapy were excluded due to confounding intervention variables such as concurrent chemotherapy or other therapies that may have influenced the outcomes. Supplementary materials of these studies were evaluated for possible subgroup analyses of the radiotherapy-only patients; however, there were insufficient data for inclusion. Certainly, more trials are needed to evaluate the potential clinical outcome radio-protective effects of exercise in patients undergoing radiotherapy.

We hypothesize that exercise may have radio-protective effects due to molecular effects through cellular adaptations: increased nutraceutical availability to attenuate oxidative stress, increased antioxidant activity, increased DNA repair capacity, and increased resistance against ROS/inflammatory damage (22, 67). However, to date, there are no human trials linking radioprotection and exercise directly (68). This review found a positive effect in favor of exercise vs. no exercise in the context of radiation exposure, on all levels of biological organization (macro-molecular, cell/tissue, and organ/system). The presented data suggest that both aerobic and resistive exercise performed before, during, and after irradiation increases antioxidant activity and DNA repair, followed by reduced inflammation and reduced DNA damage. At cellular and tissue levels, positive effects of exercise with respect to radiation were manifested by restoring precursor cell and neurogenesis levels, as well as increased bone mineral density, hematopoietic stem cell rescue, and progenitor cell counts (HSPC). Furthermore, at the organ's and system's levels, physical training reduced radiation-induced fatigue, organ toxicity and cognitive function impairments. Given the heterogeneous set of articles and insufficient outcome measures, more systems biology studies are required to explain how physical training counteracts the detrimental effects of IR mechanistically. Nevertheless, the overall balance of evidence points toward a radio-protective effect of physical exercise.

There is a paucity of evidence from which to evaluate how exercise modality, duration, intensity, and frequency may influence radio-protective effects. Such investigations are urgently needed in order to determine the optimal approach for exercise as a radio-protective tool. In addition, the timing, type, and dosage (dose and dose rate) of radiation exposure in relation to exercise may have an effect in the outcomes relating to radioprotection. This is particularly important as the studies reported in this review point to a high degree of heterogeneity in terms of the timing, type, and dose of radiation used to study IR effects. Currently, based on the present data, the variability or modality of exercise as it relates to radiation dose, type, or timing do not point to a specific link, but it would be valuable if further research would investigate this relationship.

### Applicability to Terrestrial Medicine

Since cancer survival is linked to better treatment outcomes and reduced side effects of chemotherapy and radiotherapy, exercise may prove to be a valuable adjunct to cancer treatments (69). Moreover, exercise is potentially relatively easy to implement and could be a potential therapeutic adjunct for a range of oncologic patients undergoing radiotherapy—to improve not only clinical outcomes but also quality of life including fatigue reduction (Figure 5). Moreover, exercise training could also help reduce radiation-induced cancers, as secondary tissue effects are a significant concern in oncology (70). In fact, it was previously reported that performing moderate physical activity was associated with protection against radiotherapy-induced cardiac disease (21). Exercise training has also been shown to be very safe even in patients of low functional and high disease burden states such as oncology patients (52, 71, 72).

The present review suggests that aerobic exercise may mitigate the detrimental effects of carbon ion exposure in rats (Figure 6) and thus appears to be a promising candidate for reducing the negative effects of HZE particle irradiations with uprising hadron cancer therapies. These findings may be applicable to aid in the reduction of secondary tissue damage from radiation and to improve overall clinical radiotherapy outcomes (73). Additionally, with newer radiation therapies, it is crucial to further our understanding of biological countermeasures to improve patient outcomes and prevent side effects (1).

As the present literature search did not include any studies with exercise and low-dose and low-dose-rate exposures, it is difficult to draw conclusions for occupational radiation hazards. However, much of the cellular adaptations seen with exercise as reported in this systematic review with decreased DNA damage, decreased inflammation, and increased antioxidant activity could have a radio-protective effect in long-term exposures of radiation workers. It is also reported that physical exercise is an effective preventive measure to reduce disease incidence and recurrence risk, as part of public health strategies (74).

Although the results of this systematic literature review are encouraging and warrant further research in this field, the data are insufficient to directly inform clinical practice. It is therefore recommended that future research should consider the elements of risk of bias in designing and conducting studies and provide more details in reporting risk of bias and results.

### Applicability to Space Medicine

Although radiation is one of the main hazards that need to be overcome in planning for deep space missions, there are currently no effective and practical approaches to protect astronauts (4). Engineering countermeasures do not exist for complete radiation protection mainly due to the lack of technologies and the current insurmountable engineering challenges of launching enough shielding material to protect against the deep space radiation environment (4, 75, 76). In light of this, biological countermeasures and other ways to adapt to the deep space radiation environment need to be further considered. The current understanding of the complex space environment and how human biology interacts with it is an area of active research that can be combined with new countermeasures such as exercise (4, 77).

Currently, exercise countermeasures are used on the International Space Station (ISS) as a part of regular operations to protect against various negative adaptations to microgravity (78). It is currently not known if physical exercise could effectively mitigate the detrimental effects of space radiation environment to human body, however the present findings suggest a beneficial potential of physical activity. It could be of particular importance in deep space missions, as astronauts will be exposed to higher absolute doses and dose rates, with a higher portion of HZE particles from GCR, compared to LEO. Thus, we need to further develop both biological and engineering countermeasures against deep space radiation. We showed that physical training has a potential to mitigate radiation damage from HZE ions; however, more studies are needed specifically to assess the extent to which it could reduce health risks of astronauts exposed to the space radiation environment.

It should also be noted that space radiation is complex in modality and large fluctuations of dosage depending on solar activity and shielding efficacy are normal in the hostile deep space environment. Radiation dose estimations for a 6-month ISS mission and a 1,000-day Mars mission are provided in Table 2. However, most of the studies that were analyzed refer to a single type of radiation at a specified (high) dose during a specific time interval. Thus, caution must be taken to apply those findings to the deep space environment as exposure to complex radiation field in deep space can activate both cytoprotective and cytodestructive pathways (81). For example, the knowledge about HZE particles and how they interact with tissues is incomplete. Although HZEs occur in low flux, the high-energy deposition along the particle track can have severe damaging effects (68, 82). Fukuda et al. (54) seem to point into the direction that physical exercise could protect from HZE-induced damage (Figure 6); however, radiobiological research is only beginning to look at the complex space radiation environment in considering the different energies and types of radiation that exist in deep space (83). There is also the requirement for improved deep space radiation ground-based analogs to study the effects of complex deep space radiation on living organisms.

**Table 2:** Estimated radiation exposure during space missions.

**Mission type**	Dose rate^*^ (μSv/h)	**Mission duration**	**Radiation dose (mSv)**	**References**
ISS	22–27	180 days	95–120	(16, 17, 79)
Mars	Free space: 77 Mars surface: 26	1,000 days	1,060	(15) (80)

* *Depends on solar activity and shielding/flight altitude of ISS*.

In conclusion, this systematic review suggests a clear general beneficial effect of exercise when either animals or humans are exposed to IR in the context of DNA damage, antioxidant activity, inflammation, neurogenesis, cellular function, and clinical and functional outcomes. Although the 22 studies analyzed in this review are heterogeneous in terms of population and outcomes, most studies demonstrated positive effect sizes in favor of physical exercise having a radio-protective effect. Further studies are however required to conclude the extent and most beneficial modality of exercise as a countermeasure against IR as an adjunct for both terrestrial clinical radiotherapy and human space exploration.

## Data Availability Statement

The original contributions generated for this study are included in the article/Supplementary Material, further inquiries can be directed to the corresponding author/s.

## Author Contributions

DK performed literature search and screening, data extraction, effect size calculations, and wrote the manuscript. TW supported literature screening and reviewed the manuscript. US reviewed the manuscript. CH consulted on merits, reviewed and edited the manuscript, and checked the proofs. MN consulted on methodology, reviewed and edited the manuscript. DG reviewed the manuscript. AF supported every stage of the project, including active literature screening, extracted metadata, created the figures and tables, and wrote the manuscript. All authors contributed to the article and approved the submitted version.

## Conflict of Interest

TW and DG were employed by the company KBR GmbH. The remaining authors declare that the research was conducted in the absence of any commercial or financial relationships that could be construed as a potential conflict of interest.

## Supplementary Material

Supplementary Table 1 Boolean search operators and terms used in the systematic review. Includes all Boolean search logic, categories, keywords, and search masks that were used in the main literature search.

Supplementary Table 2 List of the outcomes with calculated effect sizes, and additional meta-data from all the studies included in the systematic review. It was impossible to calculate effect sizes for two outcomes and they are indicated in gray.

Supplementary Table 3 Summary of risk of bias and quality. Each of the 22 articles included were assessed for the risk of bias and its methodological quality. “+” indicates low risk, “-” indicates high risk, “?” indicates unclear risk, and “NA” indicates assessment was not applicable.

Supplementary Table 4 Outcome parameters unable to be plotted on an effect size plot.
